# Walking (and talking) the plank: dual-task performance costs in a virtual balance-threatening environment

**DOI:** 10.1007/s00221-024-06807-w

**Published:** 2024-03-27

**Authors:** Tiphanie E. Raffegeau, Sarah A. Brinkerhoff, Mindie Clark, Ashlee D. McBride, A. Mark Williams, Peter C. Fino, Bradley Fawver

**Affiliations:** 1 George Mason University, School of Kinesiology, 10890 George Mason Circle, Katherine Johnson Hall 201G, MSN 4E5, Manassas, VA 20110 USA; 2 Department of Health and Kinesiology, University of Utah, Salt Lake City, UT USA; 3 Department of Health and Human Performance, Rocky Mountain College, Billings, MT USA; 4 Walter Reed Army Institute of Research-West, Joint Base Lewis-McChord, Tacoma, WA USA; 5 Institute for Human and Machine Cognition, Human Health, Resilience and Performance, Pensacola, FL USA

**Keywords:** Anxiety, Cognition, Divided attention, Fear of falling, Mobility, Speech

## Abstract

We evaluated the effects of engaging in extemporaneous speech in healthy young adults while they walked in a virtual environment meant to elicit low or high levels of mobility-related anxiety. We expected that mobility-related anxiety imposed by a simulated balance threat (i.e., virtual elevation) would impair walking behavior and lead to greater dual-task costs. Altogether, 15 adults (age = 25.6 ± 4.7 yrs, 7 women) walked at their self-selected speed within a VR environment that simulated a low (ground) and high elevation (15 m) setting while speaking extemporaneously (dual-task) or not speaking (single-task). Likert-scale ratings of cognitive and somatic anxiety, confidence, and mental effort were evaluated and gait speed, step length, and step width, as well as the variability of each, was calculated for every trial. Silent speech pauses (> 150 ms) were determined from audio recordings to infer the cognitive costs of extemporaneous speech planning at low and high virtual elevation. Results indicated that the presence of a balance threat and the inclusion of a concurrent speech task both perturbed gait kinematics, but the virtual height illusion led to increased anxiety and mental effort and a decrease in confidence. The extemporaneous speech pauses were longer on average when walking, but no effects of virtual elevation were reported. Trends toward interaction effects arose in self-reported responses, with participants reporting more comfort walking at virtual heights if they engaged in extemporaneous speech. Walking at virtual elevation and while talking may have independent and significant effects on gait; both effects were robust and did not support an interaction when combined (i.e., walking and talking at virtual heights). The nature of extemporaneous speech may have distracted participants from the detrimental effects of walking in anxiety-inducing settings.

**Supplementary Information:**

The online version contains supplementary material available at 10.1007/s00221-024-06807-w.

## Introduction

Mobility in daily life often requires managing concurrent cognitive and motor demands under conditions that can threaten balance, such as talking to a friend while navigating a busy crosswalk. Published reports describe how interference between cognitive and motor processes influence mobility behavior under various environmental and contextual demands (Woollacott and Shumway-Cook 2002; Yogev-Seligmann et al. 2012). Scientists have specifically sought to understand how individuals manage simultaneous perceptual-cognitive demands during locomotor tasks in young adults (Siu et al. 2008; Raffegeau et al. 2018), older adults (Holtzer et al. 2011; Beurskens and Bock 2013; Beurskens et al. 2014), in people with deficits in cognitive-motor function (Montero-Odasso et al. 2017; Holtzer and Izzetoglu 2020; Li and Harmer 2020), and patients with movement disorders like Parkinson’s (Camicioli et al. 1998; Baker et al. 2007; Beck et al. 2018). Most studies in this growing body of literature are focused on factors influencing fall-risk, comparatively, less is understood about individual differences in environmental risk-assessment and the reciprocal influence of mobility-related anxiety on cognitive-motor behaviors (Yogev-Seligmann et al. 2012; Young and Williams 2015).

Theoretical frameworks that address the hierarchical nature of dual-tasking during gait suggest that healthy people use a ‘posture-first’ strategy by focusing primarily on their gait performance in hazardous situations where balance is perceived to be at risk (Yogev-Seligmann et al. 2012). The integrated prioritization model further implies that individual differences in relative motor (i.e., ‘postural reserve’) and cognitive capabilities (i.e. ‘cognitive reserve’) dictate the allocation of perceptual-cognitive resources during gait (Yogev-Seligmann et al. 2012). Attentional Control Theory (ACT) provides an alternative framework to understand how performance-related state anxiety influences perceptual-cognitive control (Eysenck et al. 2007; Young and Williams 2015). ACT specifies that anxiety disrupts attentional control by increasing the bottom-up ‘distraction bias’ to external threats in lieu of processing goal-directed (i.e., task-relevant) information (Eysenck et al. 2007; Young and Williams 2015). When applied to walking, ACT predicts that an individual undergoing acute fall-related anxiety would likely disengage from any additional cognitive demand as perceptual-cognitive resources are already strained by the addition of worrisome thoughts about performance, such as self-preoccupation and concerns about performance evaluation (Young and Williams 2015). Within the context of posture and gait behavior, other related frameworks such as Conscious Processing Theory (Masters and Maxwell 2008) operationalize disruptions in attentional and motor control as ‘reinvestment,’ which entails devoting resources to a task that was previously autonomous. Reinvestment can lead to sub-optimal cognitive and sensory functioning during walking (Young et al. 2016; Uiga et al. 2018; Ellmers et al. 2020), as well as promoting a ‘self-’ or ‘internal-focus’ (Wulf 2013) that can inhibit processing of external stimuli during complex mobility tasks for older adults (Ellmers et al. 2020; Kal et al. 2022). Current theories suggest that cognitive (e.g., a dual task) and psychological demands (e.g., brought on by balance threat) draw from the same limited pool of resources. Competition for shared resources is thought to lead to rigidly controlled motor behavior (e.g., reinvestment in controlled processes, internal focus), or distraction from motor skill execution (e.g., through task-irrelevant thoughts, increased sensitivity to external stimuli, Wulf 2013; Young et al. 2016; Uiga et al. 2018; Ellmers et al. 2020; Kal et al. 2022).

A well-practiced cognitive task that does not directly compete with sensory integration may elicit different effects on cognitive-motor control in stressful environments, but most researchers have typically relied on observations from tasks that might be influenced by age-related declines in sensory interference, such as an auditory reaction time task (Nnodim et al. 2016) or a visuospatial distractor (i.e. clock-monitoring; Plummer-D’Amato et al. 2011). In healthy adults, published reports have highlighted conflicting sensorimotor goals between auditory (Siu et al. 2008; Worden and Vallis 2014) or visually demanding (Kimura and van Deursen 2020) cognitive tasks and visual integration for gait. To avoid sensory interference, many researchers have used the serial subtraction task (i.e., subtract from 100 by 3 or 7; Lindenberger et al. 2000; Schaefer et al. 2015) which challenges cognitive processes but is subject to biases associated with socio-economic background or education levels (Birnie et al. 2011) and can impose a rhythmicity on gait that could influence walking performance (Yogev et al. 2005; Penati et al. 2020). Moreover, contrived cognitive tasks have a ‘purity’ problem, in which a targeted cognitive process engages broader network-wide processes (Miyake et al. 2000). Such tasks may serve as distractions that bias attentional control more than well-practiced cognitive demands, particularly in anxiety-inducing settings. Finally, due to learning effects (Lovett 2005), there is concern over whether results derived from previous studies that average performance across multiple trials represent realistic cognitive-motor behavior that does not involve repeated practice.

Alternatively, the social consequences of extemporaneous speech production might encourage healthy adults to prioritize talking behavior over gait performance (Raffegeau et al. 2018). The challenge involved in concurrent walking while talking is highlighted in studies which report that older adults at risk of falling must stop walking to continue talking (Lundin-Olsson et al. 1997). In healthy adults, we have previously observed that only when the demands of a motor task become too difficult (i.e. avoiding an obstacle) do healthy people demonstrate a trade-off between concurrent extemporaneous speech and complex locomotion; allowing costs to speech production in favor of dedicating resources to motor performance towards a demanding locomotor task (Raffegeau et al. 2018). It is feasible that walking while talking in contexts that elicit mobility-related anxiety in healthy young adults would result in different resource allocation patterns than those previously observed using laboratory-based tasks. By examining the single and dual-task costs associated with well-practiced cognitive demands under conditions of low and high perceived threats to mobility in healthy adults, we distinguish the influence of relevant cognitive demands on attentional biases in anxiety-inducing settings.

In the present study, we build on existing theoretical frameworks to examine how individuals manage cognitive-motor demands in situations that elicit anxiety about gait performance. Specifically, we used a previously validated virtual reality (VR) based approach (Raffegeau et al. 2020a) to induce state anxiety with a simulated high elevation environment that mimics traditional laboratory-based methods of physically lifting people to high elevation (Cleworth et al. 2012; Adkin and Carpenter 2018). Healthy young adults walked alone (single-task) and walked while performing a concurrent extemporaneous speech monologue (dual-task) in virtual low and high elevation settings. We predicted, based on similar studies in the field (Cleworth et al. 2012; Adkin and Carpenter 2018; Raffegeau et al. 2020a, b, 2023) and prevailing theoretical frameworks (Young et al. 2016; Ellmers and Young 2018; Ellmers et al. 2020), that walking at virtual elevation without a concurrent cognitive task would be associated with more ‘protective’ walking behavior (i.e., slower gait speed and shorter and wider steps to avoid potential balance perturbations) compared to simulated ground level walking. Since extemporaneous speech is cognitively demanding, but does not directly conflict with sensory demands during walking, we predicted that healthy adults would not demonstrate a ‘tradeoff’ between cognitive and gait performance. We alternatively predicted that healthy young participants would preserve their speech performance within anxiety-inducing settings, even as gait behavior became more conservative. Conversely, if healthy adults allowed both cognitive and motor performance to decline while walking in anxiety-inducing settings, it would suggest that balance threat leads participants to dedicate resources to prevent a potential fall. We predicted, based on ACT (Eysenck and Calvo 1992; Eysenck et al. 2007), that at virtual ground level (i.e., without a balance threat), young adults would prioritize the extemporaneous speech task and exhibit compensations in gait behavior as compared to walking without the cognitive dual-task (i.e., slower gait speed, shorter and wider steps). However, at virtual high elevation, we expected performance costs brought on by greater levels of mobility-related anxiety would be reflected in gait and speech outcomes, such that individuals would both adopt conservative gait behavior (i.e., slower gait speed, shorter and wider steps) and exhibit interference in speech performance (i.e., more frequent speech pauses of greater duration).

## Methods

### Participants

Participants were recruited using a convenience strategy, yielding a sample of 15 relatively young healthy adults (mean age = 25.6 + 4.7 yrs, 7 women). Individuals were included if their vision was normal or corrected to normal, they had no orthopedic injuries causing discomfort during walking, and English was their primary language. No participant reported experiencing a fall in the previous six months, defined as ‘coming to a lower level unintentionally’ (Montero-Odasso et al. 2022). All participants provided informed consent using a protocol approved by the local Institutional Review Board.

### Instrumentation and task

### Virtual gait task

**Fig. 1:**
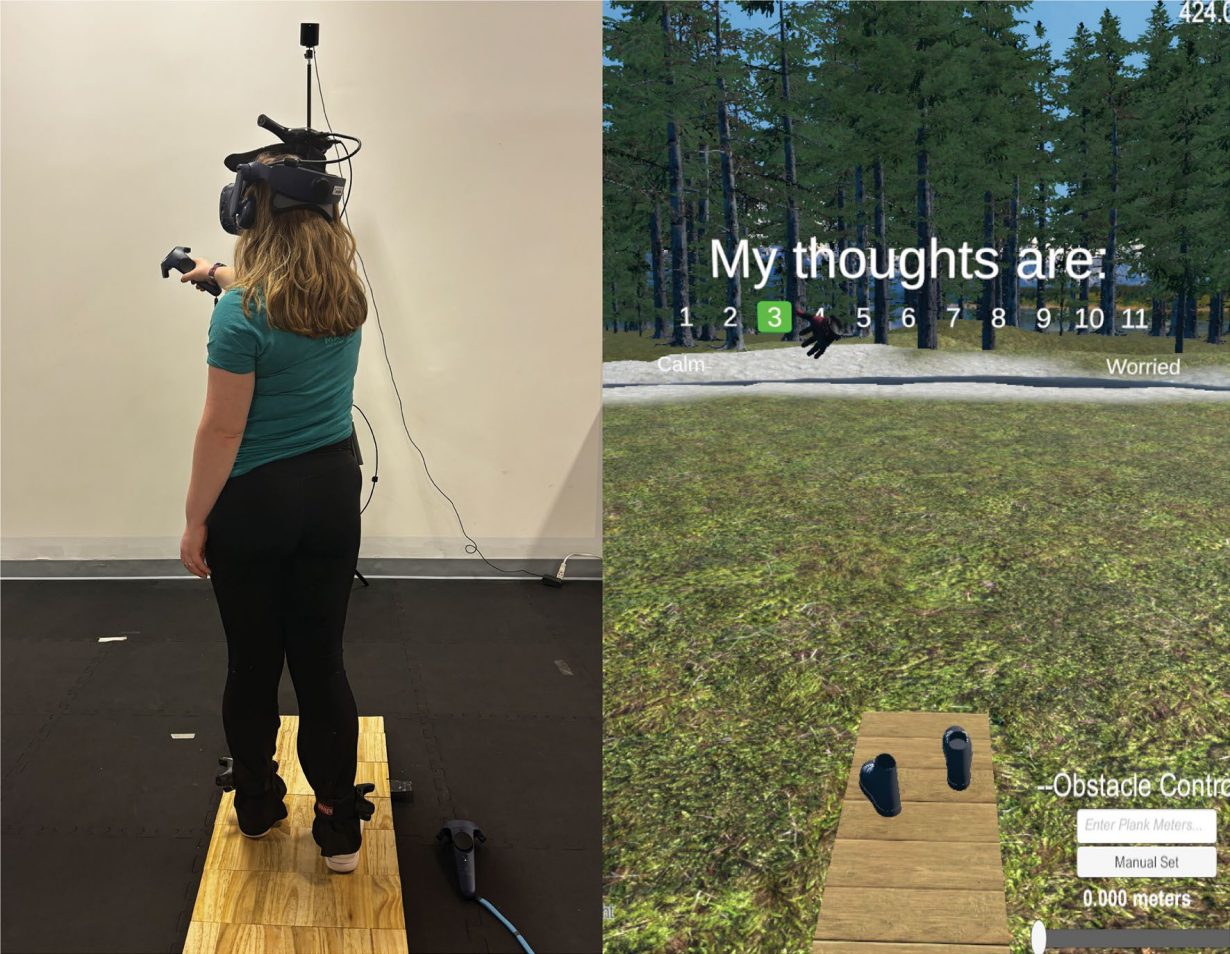
After each condition, participants used a hand controller to select their responses for each self-report item. Likert scales were presented in the virtual environment to determine the participants level of somatic and cognitive (shown here) anxiety and confidence (Mental Readiness Form; MRF-3, Krane 2016), as well as their level of mental effort devoted to task completion (Rating Scale for Mental Effort; RSME, Zijlstra 1993)

Based on established methods (Raffegeau et al. 2020a, 2023), participants wore the HTC Vive (version 2.0, Bellevue, WA, USA) immersive head mounted display (HMD) displaying a 0.4 × 5.2 m virtual walkway matched to a real path. The global 3D coordinates for each corner of the walkway in the virtual space were determined by capturing the position of each corner of the physical walkway using a hand controller, matching the dimensions and coordinates of the real-world walkway to the virtual simulation. In accordance with International Society of Biomechanics Standards, the center and starting point of the walkway was defined as the origin, x was the sagittal path of progression, z was the mediolateral axis, and y was the vertical axis (International Society of Biomechanics Standardization and Terminology Committee 2002). Participants wore trackers around their ankles (HTC Vive version 2.0, Bellevue, WA) to provide ongoing visual feedback of where their feet were in the virtual space. For two minutes, participants explored the virtual space and were allowed to walk or stand along the walkway in their preferred fashion (Fig. 1).

### Extemporaneous speech task

Participants were provided with a list of 26 conversation topics (e.g., first job, favorite TV show, recent trips taken) and asked to choose six of those topics they could talk about for at least one minute. Extra topics were selected to prevent topic bias from the participant so that no speech sample was based on a particularly vivid or easy-to-remember monologue, and in case of recording difficulty, we could redo the single task condition. At the beginning of each extemporaneous speech trial, participants were randomly assigned one of their selected topics to speak about. After the participants were assigned the topic but before they began speaking, they were given a short time (5–10 s) to think about what they would talk about before the trial began. Participants first completed the extemporaneous speech task while seated in the laboratory space, representing single-task cognitive performance. The instructions emphasized that ‘what you say doesn’t matter, just that you can keep talking the entire time’.

### Procedure

Each data collection session began with the participant completing a series of surveys and cognitive tests to address dispositional differences (Table 1). Virtual walking conditions (i.e., blocks) were pseudorandomized so that the order of single vs. dual task conditions were always counterbalanced across participants. The virtual low elevation environment was always presented first in each single or dual task condition to ensure participants experienced the largest effects of mobility-related anxiety at virtual high elevation (1st Block = Low, 2nd Block = High). Participants walked continuously at their self-selected pace for one minute, completing between 7–12 passes on the 5.2-m walkway consisting of 3–5 strides per pass. To transition between low and high virtual elevation, participants were seated in a chair facing forward at the beginning of the walkway with their feet on the path and eyes looking straight ahead as they were lifted to an approximate 15-m above ground at the rate of a standard elevator (1 m/s). During walking trials, participants were instructed to ‘stay on the path and walk without speaking at their comfortable pace and continue walking until they heard ‘stop’. During dual-task trials, participants were instructed to ‘walk and talk like you were speaking to a friend’ with no additional instructions to prioritize one task over another. To examine participants’ perceptions, four rating scales (Sect. “Perceptions of performance”) were presented inside the headset for completion using a game controller that was handed to participants after each walk was complete (Fig. 1). Instructions emphasized participants should reflect on the walking experience he/she had just completed, and were delivered identically by the same researcher for all participants to ensure consistency.

**Table 1:** Participant characteristics (*N* = 15)

	Mean	*SD*	Range	
			Lower limit	Upper limit
Age (y)	25.6	4.7	22.63	27.81
Height (cm)	168.0	10.7	161.5	173.0
Mass (kg)	68.5	12.5	59.7	77.1
Leg length (mm)	92.8	6.7	87.4	97.4
STAI-T (score)	31.4	4.6	31.0	38.2
STAI-S (score)	25.2	5.2	23.0	33.2
Stroop Congruent (score)	93.6	8.1	86.5	99.5
Stroop Incongruent (score)	75.6	14.5	69.5	85.5
TMT-A (s)	17.3	5.1	12.9	21.0
TMT-B (s)	34.9	14.3	29.2	43.3

*SD *standard deviation, *y* years, *cm* centimeters, *kg* kilograms, *mm* millimeters, *STAI-T/S* State-Trait Anxiety Inventory, Stroop scores represent the number of correct responses per 45 s, *TMT-A/B* Trail Making Test A and B, *s* seconds

### Measures

#### Dispositional anxiety

Anxiety was assessed using the State-Trait Anxiety Inventory (STAI; Spielberger 1983). Scores range from 20 to 80, with higher scores reflecting greater trait (i.e., generally) or state (i.e., day of the study) anxiety.

#### Cognitive function

Participants completed two common clinical tests of cognition (i.e., executive functioning) on paper, the Stroop Task and the Trail Making Test, both of which have been shown to be related to cognitive-motor performance and mobility (Hobert et al. 2011; Raffegeau et al. 2019). Our version of the Stroop Task consisted of two parts: (1) Congruent, a test of response time, requiring participants to name as many colored letters (e.g., “XXXXX”) as possible within 45 s (measuring visual scanning and response time); and (2) Incongruent, a measure of response inhibition (measuring executive function maintenance and set switching), requiring participants to name as many ink colors of mismatched color-words in 45 s (Jensen and Rohwer 1966; Arbuthnott and Frank 2000). Three participants reported being red-blue colorblind and did not complete the Stroop tests. Our version of the Trail Making Test took place in two parts as well. For Part A, participants were required to connect dots containing only numbers in ascending order, a measure of executive attention. For Part B, participants were required to connect dots that alternated from numbers to letters in ascending order, a measure of executive switching and inhibition (Sánchez-Cubillo et al. 2009). If participants made an error, they were immediately informed and were instructed to correct themselves before completing the puzzle.

#### Perceptions of performance

We used validated Likert scales to capture self-reported perceptions of performance. The Mental Readiness Form-3 (MRF-3; Krane 2016) was administered to assess cognitive (i.e., worry) and somatic (i.e., arousal) components of anxiety experienced during each condition, as well as participants’ level of confidence in their ability to complete the task, using an 11-point Likert-scale. Cognitive anxiety ratings (i.e., worrying thoughts) were prompted with the root statement ‘my thoughts were’, with responses ranging from 1, ‘very calm,’ to 11, ‘very worried.’ Somatic anxiety ratings (i.e., moment to moment changes in physiological arousal) were prompted by the root statement ‘my body feels,’ and ranged from 1, ‘very relaxed,’ to 11, ‘very tense.’ Participants self-rated confidence in their ability to complete the task was evaluated with the root statement ‘I am feeling,’ and response ranging from 1, ‘very confident,’ to 11, ‘not confident at all’. For analysis, we reverse-scored this measure so that higher values would indicate greater levels of confidence in their ability to complete the task. Participants indicated the level of mental effort required to complete the task in each condition using the Rating Scale of Mental Effort (RSME; Zijlstra 1993) which is a 0–150 scale that ranges from ‘0: absolutely no effort’ to ‘150: extreme effort.’

#### Gait kinematics

Step length, step width, and gait speed, and the variability (standard deviation) of each were calculated using a custom MATLAB script (version R2022b, Natick, MA, USA) by using the linear position between the HMD and the two ankle tracking accessories placed on the lateral aspect of participants’ ankles (see Data Processing). The HTC Vive collects variable position sampling rates (e.g., one trial frame rate range = 89 Hz to 93 Hz) depending on the relative speed of motion and the independent sampling rates of the lighthouses and tracker accessories (Niehorster et al. 2017). Consequently, we resampled the data to 100 Hz using the *resample* function with linear interpolation in MATLAB. Errors in position tracking were identified by removing erroneous position data that were recorded below the origin (the walkway) and replacing resultant missing data with spline-filled data points. The spline-filled data were then filtered with a zero-lag fourth order low pass Butterworth filter (6 Hz). Foot contacts were identified as peaks in the vector between the HMD and each foot tracker. The 3D position of the feet and time at each identified peak was extracted at foot strike (Zeni et al. 2008).

Straight steps were isolated by retaining the steps taken within the central 4.4-m portion of the walkway and removing turning steps from the analysis. However, some individuals did not walk the entire length of the walkway, especially at virtual high elevation. Therefore, for each individual, we calculated their maximum distance travelled on the walkway and extracted steps within 0.5 m from that individual’s maximum distance. Step length was calculated for each foot contact as the absolute distance between the ankle-worn sensors in the anterior posterior direction, and step width was the absolute distance between the ankle-worn sensors in the mediolateral direction for successive steps. Gait speed was calculated as step length divided by the time between two consecutive footfalls. Left and right step values were averaged to represent overall gait performance. Variability was calculated as the standard deviation across steps.

#### Speech performance

The participant was fitted with a wireless microphone (Lavalier, model WMX-1) to record speech. The frequency and duration of silent speech pauses are interpreted as indicators of cognitive costs incurred in each extemporaneous speech condition (Lee et al. 2019; Darling-White and Huber 2020). Previous work has demonstrated speech pauses during an extemporaneous speech monologue are sensitive to motor difficulty (Raffegeau et al. 2018). Seated extemporaneous speech is considered the baseline for cognitive performance capacity (single-task). The number and mean duration of pauses during the extemporaneous speech task (silent pause > 150 ms) were identified by a trained research assistant using open-access software (PRAAT, v 6.2.14). Based on previous methods (Raffegeau et al. 2018; Darling-White and Huber 2020) research assistants marked the beginning and end of silent pauses using spectrograms and waveforms. A custom MATLAB code determined pause length, the number of pauses, and the total pause time within a trial.

### Statistical analyses

We used the *fitlme* and *anova* (ANOVA; analysis of variance) functions in MATLAB to analyze linear mixed-effect regression models and type III tests for fixed effects, respectively. Separate, fully factorial linear mixed-effect regressions (LMERs) were used to evaluate the effect of Height (low vs. high) and Cognitive Demand (single vs. dual) on gait performance (i.e., gait speed, step length, step width, and their variability). Models included a fully crossed random intercept by participant, participant within Height, and participant within Cognitive Demand, thereby accounting for within-participant variance at each elevation and cognitive function. Height was reference-coded such that low elevation was the reference (Height: low = 0, high =  + 1). Cognitive Demand was reference-coded such that single-task was the reference (cognitive demand: single-task = 0, dual-task =  + 1). Therefore, the reported unstandardized *β* (beta) weights and respective confidence intervals [CI] can interpreted as mean differences between factor levels, and interaction effects would represent change due to virtual elevation multiplied by the change from single to dual task. ANOVA *F*-scores represent a standardized relative effect for each model. All model outputs are provided in the Supplementary Appendix (Supplementary Data Table S1–S10).

Self-reported ratings were analyzed using fully factorial LMERs to determine the effect of Height (low = reference vs. high) and Cognitive Demand (single = reference vs. dual-task) on perceived anxiety (somatic and cognitive), confidence, and mental effort. Although LMER is robust to violations of normality, we bootstrapped the values (*N* = 1000), sampling with replacement, to compare more robust confidence intervals in self-report ratings between low and high virtual elevation. Finally, we used mixed model ANOVAs to determine the effect of Task-Elevation (seated single task = reference vs. low DT vs. high DT) on the number of speech pauses, speech pause length, total speech pause duration. We included a random intercept of subject within condition in all models, and for speech pause length we included a random intercept and slope of subject within condition. The significance threshold for all statistical analyses was set at *α* = 0.05.

## Results

### Demographics and self-report

Out of an initial 18 participants that were evaluated, we were unable to include data from the first three due to a technical difficulty which was subsequently resolved with a slight change in the protocol related to setting up the VR system. All included participant demographics (*N* = 15, 7 women) are reported in Table 1. No substantial variability was observed among participant characteristics other than anthropometrics, Stroop Incongruent trials (i.e., response inhibition), and the Trails Making Test-B (i.e., executive function, set switching).

**Fig. 2:**
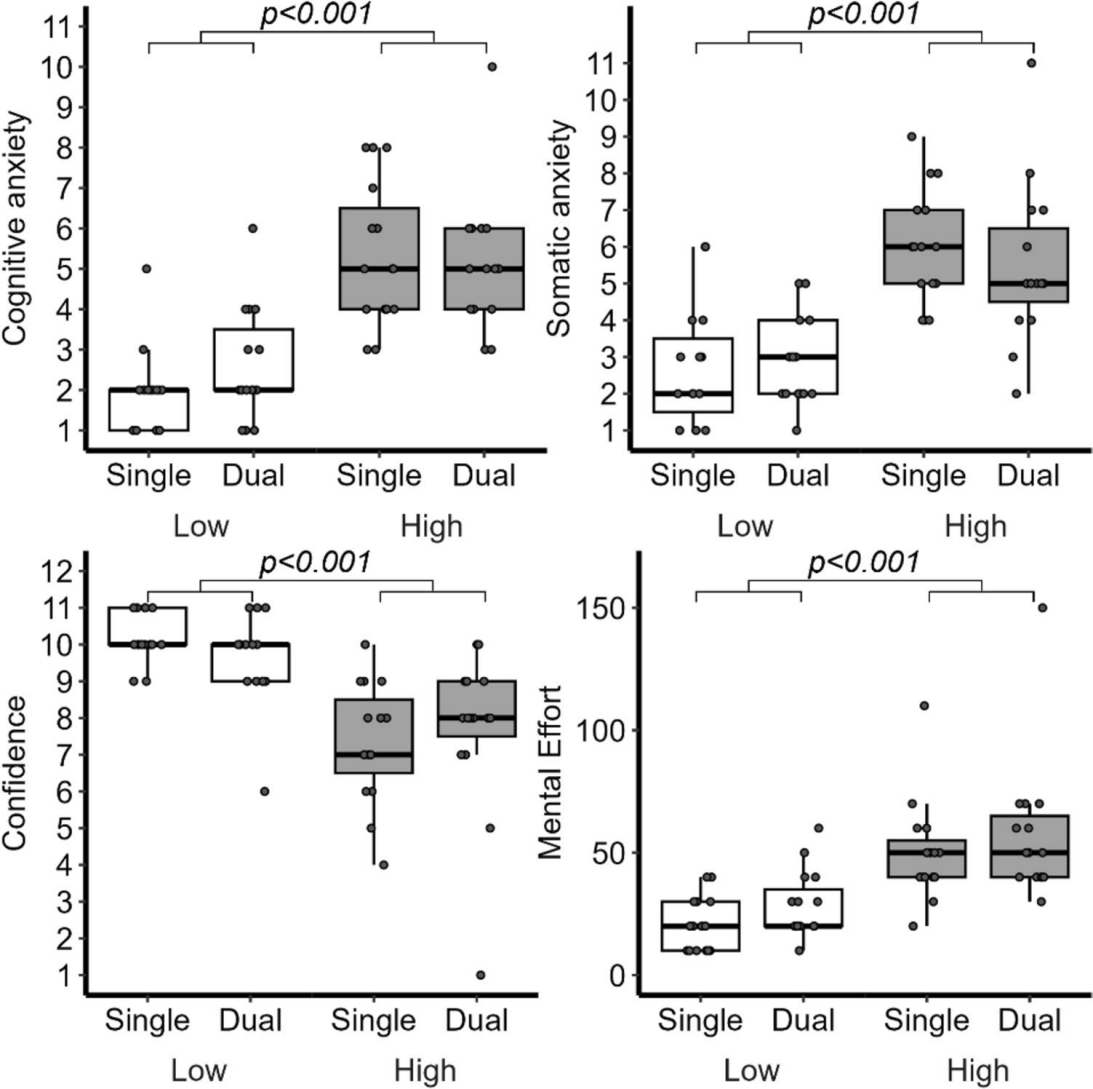
The changes in self-reported cognitive anxiety (worry; top left), somatic anxiety (tension; top right), confidence (bottom left), and mental effort (bottom left) across each walking condition

Analyses of self-reported ratings following each condition revealed statistically significant main effects of Height on participants perceptions of the cognitive, *F*(1,56) = 64.32, *p* < 0.001, and somatic, *F*(1,56) = 85.03, *p* < 0.001, components of anxiety during walking trials, as well their confidence, *F*(1,56) = 53.81, *p* < 0.001, and mental effort, *F*(1,56) = 35.60, *p* < 0.001, in executing the experimental task (see Fig. 2 and Supplementary Appendix Table S1–S4). When we decomposed these main effects, we observed that relative to the range of possible self-report response values (scored 1–11), walking at virtual elevation resulted in participants experiencing an approximate 31% increase in worrying thoughts (*β* = 3.42 [2.53, 4.32]), 30% increase in perception of changes in arousal (*β* = 3.28 [2.55, 4.02]), 26% decrease in confidence (*β* = − 2.88 [− 3.77, − 1.98]), and a 20% increase in mental effort (*β* = 29.40 [16.07, 42.72]). No main effects of Cognitive Demand (i.e., presence of a dual-task) were documented for any self-report outcome (all *p*’s > 0.111). Finally, no statistically significant interactions of Height x Cognitive Demand were observed for self-report items; however, the interaction effects for cognitive anxiety, *F*(1,56) = 10.88, *p* = 0.78, and confidence, *F*(1,56) = 18.02, *p* = 0.73, trended towards significance, suggesting that walking while talking somewhat mitigated the increases in worrying thoughts and decreases in self-efficacy experienced when walking at high elevation (*β* = − 0.89 [− 0.36, − 0.27]).

### Gait performance

**Fig. 3:**
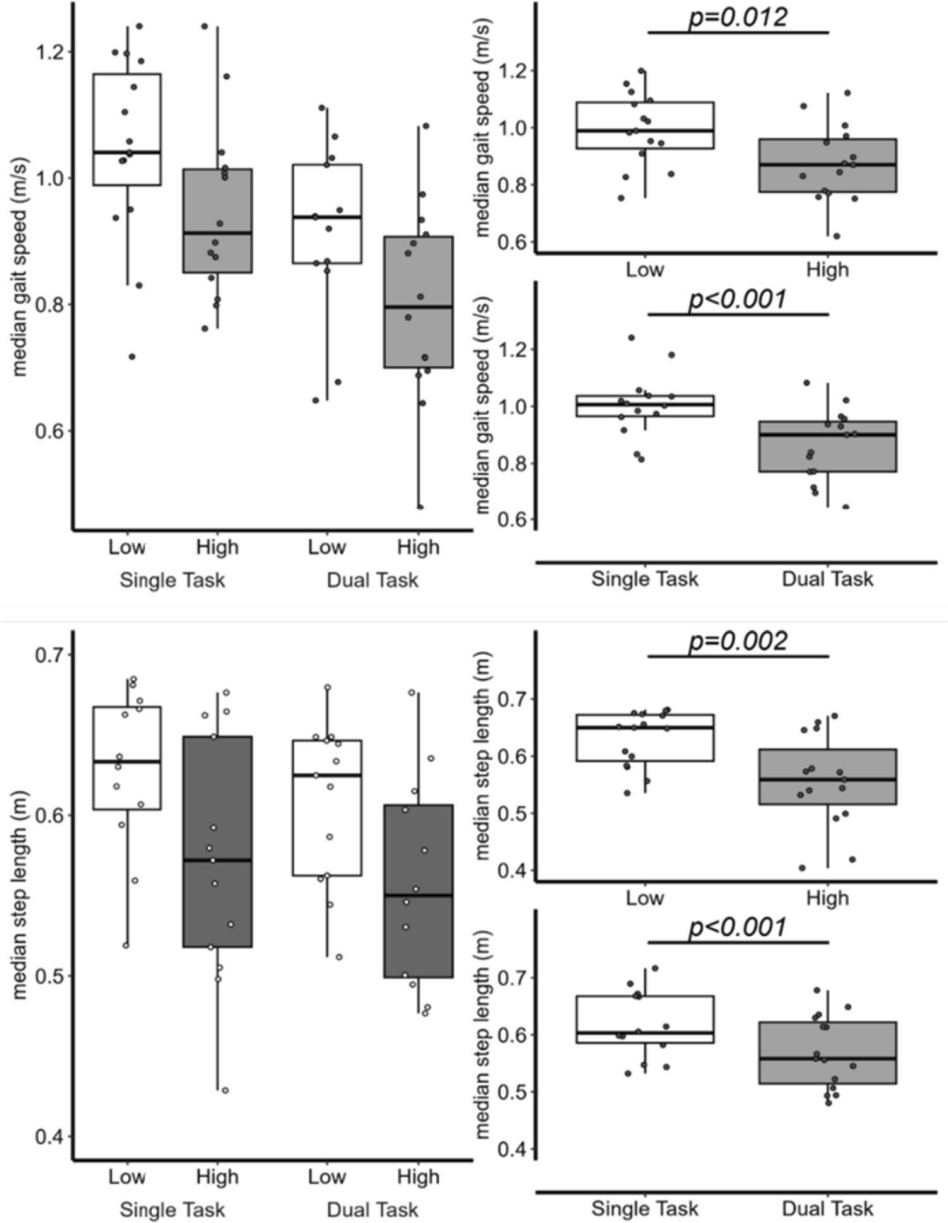
The median gait speed (top) interaction (top left) and main effects (top right) and step length (bottom) and main effects (bottom right) across virtual low versus high elevation and single versus dual-task conditions

We observed main effects of Height, *F*(1,52) = 7.15, *p* = 0.010, and Cognitive Demand, *F*(1,52) = 32.11, *p* < 0.001, on gait speed, indicating that participants walked 11% slower during the high elevation condition compared to low elevation (*β* = − 0.11 m/s [− 0.20, − 0.03]), and 14% slower during dual-task compared to single-task conditions (*β* = − 0.16 m/s [− 0.21, − 0.10]). No significant interactions were detected for gait speed (*p* = 0.951). Gait speed variability was significantly impacted by the presence of a dual-task, *F*(1,52) = 18.21, *p* < 0.001, indicating participants exhibited more consistent walking speed across trials while engaging in extemporaneous speech (*β* = − 0.05 m/s [− 0.07, − 0.03]), but no main effect of Height or interaction was revealed (*p*’s > 0.696; see Fig. 3 and Supplementary Table S5–S6).

We documented significant main effects of Height, *F*(1,52) = 10.02, *p* = 0.003, and Cognitive Demand, *F*(1,52) = 15.39, *p* < 0.001, for step length, revealing that participants shortened their steps approximately 11% during the high compared to low elevation condition (*β* = − 0.07 m [− 0.11, − 0.03]) and 7.3% during dual-task vs. single-task walking (*β* = − 0.05 m [− 0.07, − 0.02], Fig. 3). No interactions were documented for step length (*p* = 0.427) and no main effects or interactions were revealed for step length variability (all *p*’s > 0.179; see Fig. 3 and Supplementary Tables S7–S8).

**Fig. 4:**
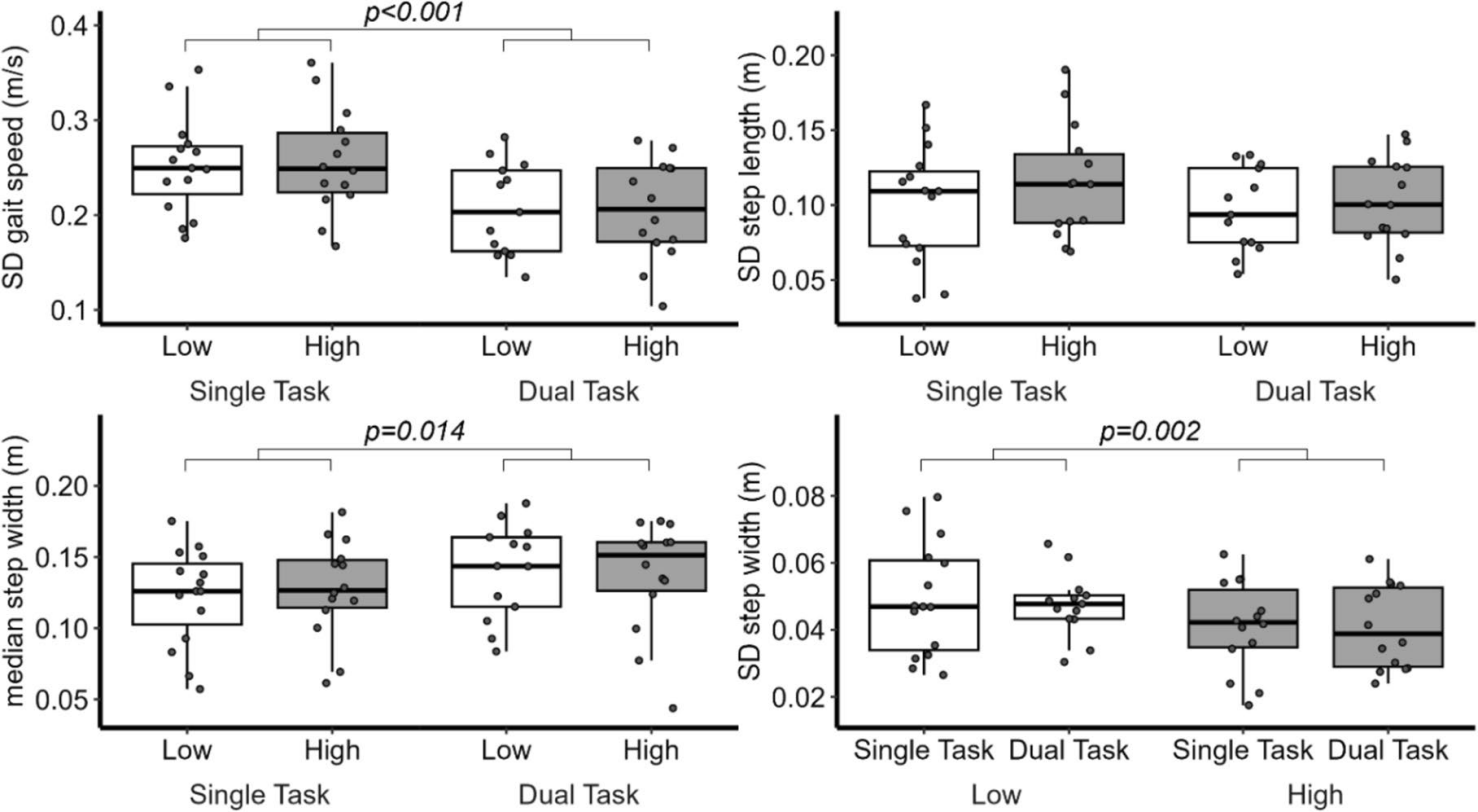
The standard deviation (SD) of gait speed (top left), step length (top right), and mean step width (bottom left), and SD step width (bottom right) for each walking condition

Models analyzing walking condition effects on step width (Fig. 4) revealed a significant main effect of Cognitive Demand, *F*(1,52) = 6.35, *p* = 0.015, with participants adopting a more conservative (i.e., 7.7% wider) stepping pattern during dual-task walking (*β* = 0.01 m [0.003, 0.02]). No significant main effects of Height or Height × Cognitive Demand interactions were observed for step width (all *p*’s > 0.301). Finally, analyses of step width variability revealed a significant main effect of Height, *F*(1,52) = 12.60, *p* < 0.001, such that participants exhibited less variable step width at high compared to low virtual elevation (*β* = − 0.009 m [− 0.01, − 0.004]), but no other main effects or interactions were observed (all *p*’s > 0.264; see Fig. 3 and Supplementary Table 9–10.

### Extemporaneous speech performance

**Fig. 5:**
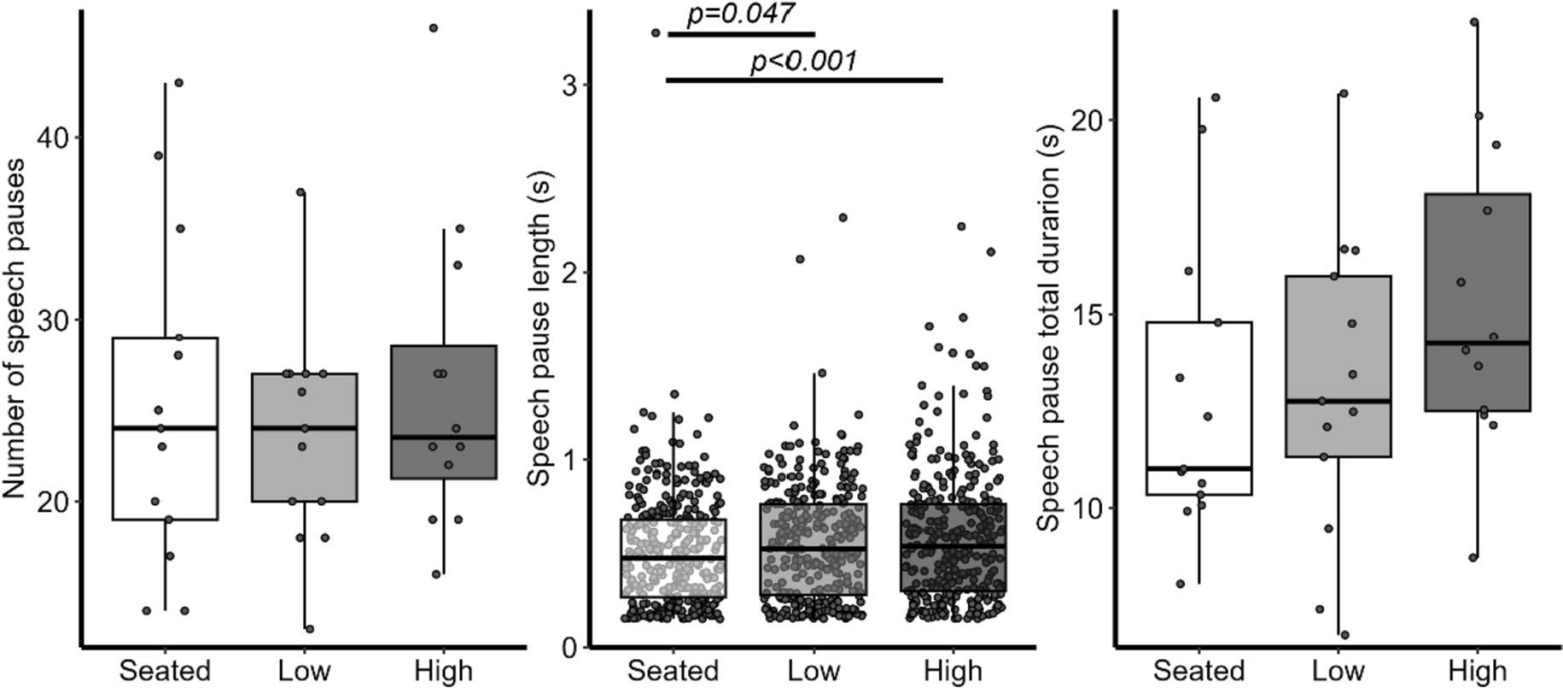
Extemporaneous speech mean silent pause number (left), length (middle), and total duration of silent pauses for each condition (right)

For the concurrent extemporaneous speech task, a main effect of Task-Elevation was observed across all silent speech pause time in seconds, *F*(2,948) = 3.719, *p* = 0.024 (Fig. 5). Follow-up planned pairwise comparisons (with Bonferroni corrections) revealed that silent speech pauses were longer while walking in the high (+ 17.8%, *p* < 0.001) and low conditions (+ 2.8%, *p* = 0.047) compared to single-task seated performance, but pauses were not significantly different between virtual low and high elevation conditions (*p* = 0.196). There were no main effects for the number of speech pauses, *F*(2,35) = 0.341, *p* = 0.714, or for the total speech pause duration, *F*(2,35) = 1.385, *p* = 0.264.

## Discussion

In the present study, we used a familiar, but challenging, cognitive task (extemporaneous speech) to examine how healthy adults managed dual-task costs during mobility in environments of varying levels of balance threat. We expected that extemporaneous speech would lead to cognitive-motor interference that would be reflected in an interaction between cognitive demands and mobility-related anxiety while participants were walking and talking at virtual high elevation. However, we did not detect an interaction between cognitive demands and mobility-related anxiety as predicted, but main effects were revealed across all performance outcomes. Findings from speech pauses (i.e., measure of cognitive interference) suggest healthy young adults prioritize talking while seated or walking, even when walking behavior is threatened by a virtual elevation. While we cannot draw any conclusions from a lack of a predicted interaction effect, future researchers should further investigate dual-task prioritization within anxiety-inducing settings. It is possible that distinct biobehavioral mechanisms are at play when walking and concurrently engaging in cognitively demanding tasks that are well-practiced and rely on different sensorimotor processes (e.g., verbal, self-generated) as the motor task (e.g., visual, proprioceptive). Gait performance was adjusted similarly to cope with cognitive demand (slower speeds, shorter and wider steps) or added mobility-related anxiety (slower speeds, shorter steps), with the exception of increases in step-width that were exclusively observed during extemporaneous speech. Taking slower and shorter steps at high elevation is in alignment with previous reports using a virtual paradigm (Raffegeau et al. 2023) and real-world heights (Brown et al. 2002; Schaefer et al. 2015).

Dual-task demands may be prioritized in some situations over walking, even when walking under conditions that threaten safety. During the extemporaneous task, participants demonstrated an increase in silent speech pauses, indicative of increased cognitive interference, from seated to walking. However, we detected no substantively greater cognitive interference as a result of state mobility-related anxiety, despite an established negative association between anxiety and speech performance reported in previous studies (Lay and Paivio 1969; Laukka et al. 2008). We argue these data indicate that participants ensured that sufficient resources were available to devote to a concurrent cognitive task when it is well-practiced like extemporaneous speech and does not interfere with the motor task, allowing cognitive-motor costs to be reflected in mobility rather than speech. Previous reports involving dual-tasks suggests young adults do not need to prioritize the gait task until motor complexity is challenged, such as during obstacle avoidance (Raffegeau et al. 2018) or walking on a narrower path (Lindenberger et al. 2000; Brown et al. 2002). Preserving extemporaneous speech during walking in healthy adults has been attributed to the social consequences of poor extemporaneous speech performance, perhaps motivating young participants to sacrifice mobility to keep speaking until they are at risk of tripping and falling (Raffegeau et al. 2018). It is possible that the high elevation environment did not challenge motor constraints enough for healthy and capable individuals to sacrifice speech in favor of their walking performance (Yogev-Seligmann et al. 2012; Raffegeau et al. 2018). Although healthy adults should have been suitably confident that they would not actually fall in the virtual environment (Young and Williams 2015), a 22% decrease in self-reported confidence in their ability to perform the task suggests virtual elevation was perceived as a more challenging walking environment. We suspect that the predicted interaction effects (i.e., task prioritization) would be more evident if the cognitive or motor demands were greater at high elevation, such as walking at a faster speed (Dennis et al. 2009; Patel et al. 2014; Callisaya et al. 2017) or avoiding an obstacle (Raffegeau et al. 2018, 2022). We also expect a population that is less confident in their capacity to perform both tasks adequately (e.g., older adults; Brown et al. 2002; Gage et al. 2003) individuals with motor impairments; Ehgoetz-Martens et al. 2015; Ehgoetz Martens et al. 2017) would demonstrate different prioritization strategies.

Self-report data from Likert scales indicated that healthy participants were more anxious, less confident, and devoted more effort towards the task when walking at simulated elevation compared to ground level and when performing the walking task while talking compared to without talking. Compared to single-task conditions, walking while talking was not associated with significant changes in self-reported anxiety, confidence, or mental effort. Although not a statistically significant effect at the a priori thresholds established, there was a trend for the interaction effect observed for cognitive anxiety (i.e., worrisome thoughts, *p* = 0.074) and self-reported somatic anxiety (i.e. perceptions of changes in arousal, *p* = 0.073) suggesting healthy adults in this sample reported less anxiety, on average, while talking at high elevation compared to walking alone, warranting further study. In contrast to our predictions, participants’ self-reported mental effort when walking at high elevation was not sensitive to the addition of a concurrent extemporaneous speech task. It is noteworthy that healthy participants reported that walking at high elevation required more mental effort than walking while talking. Since older adults with mobility impairments must stop walking to talk (Lundin-Olsson et al. 1997), future research should compare perceptions of mental effort during a well-practiced speech task across healthy adults and older people with mobility challenges.

In alignment with previous research imposing a balance threat during walking there was no effect on step width at high elevation (Gage et al. 2003; Raffegeau et al. 2023), but we detected a significant increase in step width and step width variability during the dual-task. Previously, that people take wider steps walking overground with a dual-task (Schaefer et al. 2015; Raffegeau et al. 2022), which we interpret as a result of active interference to maintain mediolateral stability during a cognitively demanding activity (Bauby and Kuo 2000). In the current study, the primary difference between walking at high elevation and walking with cognitive demand is that the balance threat encourages a narrower stepping pattern, but the dual-task leads to slower, shorter, and wider (albeit variably) steps, aligning with previous reports of young adult dual-task walking at real-world high elevation (Schaefer et al. 2015). As a result of fixed platform dimensions in the current study, increasing step width at high elevation would bring feet closer to the edge of the walkway and increase the probability of a potential fall (Raffegeau et al. 2020b). Regardless of postural threat, older adults adopt a wider step while dual-tasking at high elevation (Schaefer et al. 2015), warranting further study in an older population. While our participants coped with cognitive demands by reducing gait speed (and variability) and widening their steps, we suspect that competing goals prevented an interaction from being revealed in step width at virtual high elevation.

Given the lack of interaction effects for measures of gait performance, the results suggest that instead of a conflicting resource demand, cognitive-motor resources involved in gait may tap distinct processes when balance is threatened. The combination of cognitive-motor demands and state anxiety may not compound the deleterious effects on walking behavior in healthy young adults, particularly during conditions leveraging automaticity such as talking about a familiar topic or walking overground at a comfortable speed. Alternatively, engaging in extemporaneous speech could be a distraction from reinvestment or rumination when experiencing mobility-related anxiety. Anecdotally, participants frequently commented that “it was actually easier to walk at high heights while talking”. The interpretation that speaking was a distraction that benefitted motor performance aligns with evidence of the positive benefits of self-talk as a coping skill, even when speech is not directed at the primary motor task (Hatzigeorgiadis et al. 2009; Walter et al. 2019). Cognitive-motor demands serving as a distractor would align with existing theoretical assumptions about cognitive and attentional processes under anxiety (Eysenck et al. 2007; Masters and Maxwell 2008), as well as empirical evidence from studies using dual-task gait paradigms in healthy young (Ellmers and Young 2018) and older adults (Young et al. 2016). Focusing on the cognitive task could have allowed self-organization processes to control walking without interference, allowing gait behavior to unfold implicitly and attention to be focused externally. However, given somewhat conflicting results between self-report measures and gait performance in the present data, future researchers should include a more impaired population that would be less capable of coping with concurrent mobility-related anxiety and cognitive-motor demands.

### Limitations and future directions

In the future, researchers should aim to validate these findings among a larger and more diverse sample, as well as extending this paradigm to populations with movement impairments or psychological traits (e.g., trait anxiety) that could elicit greater sensitivity to cognitive and motor demands. Indirect measures of attentional allocation (e.g., through the use of gaze tracking) might better clarify how individuals extract information during ambulation while performing concurrent cognitive tasks. Similarly, subjective indices of attentional and motor resource allocation using recently developed self-report instruments might enhance understanding of prioritization during complex cognitive and mobility tasks (Young et al. 2020). Extemporaneous speech topics, while familiar and accessible to participants, may possess some inherent affective content (e.g., a pleasant memory of time spent with a friend, vividness of the imagery elicited by the memory, etc.). Researchers should therefore aim to explore the affective context of the speech monologue to potentially control for confounds from affect-induced changes in gait behavior (Fawver et al. 2014, 2022). Additionally, measuring lexical complexity and lexical ‘stageholders’ like filled pauses in extemporaneous speech should increase measurement sensitivity (Davie et al. 2012). Finally, the length of our walkway was extended to the limit of the virtual space to capture as many overground steps as possible, enabling us to report the variability of stepping patterns in the present study. However, removing turning steps and gait initiation/termination limited the number of steady-state steps we could include in our analysis. A treadmill-based VR paradigm may be able to capture more steady-state steps, albeit treadmill-based gait not be analogous to everyday overground gait (Row Lazzarini and Kataras 2016). As commercial VR technology improves, future researchers should investigate the variability of stepping with more consecutive steady state steps.

## Summary and conclusions

In conclusion, we extended previous work on attentional control under anxiety by testing an ecologically relevant and well-practiced cognitive task: extemporaneous speech. We successfully induced mobility-related anxiety in healthy young adults using a virtual balance threat, evidenced by decreases in self-reported confidence and increases in anxiety and mental effort. Gait kinematics indicate that, compared to ground level, walking at simulated elevation is associated with participants adopting a more conservative gait pattern (i.e., slower, shorter steps, with less variability in step width). Participants prioritized the extemporaneous speech task by walking slower (and with less variability) and taking shorter and wider steps while maintaining the length and frequency of silent speech pauses. However, no interaction effects in gait behavior were documented, suggesting that the well-practiced cognitive-motor demands of talking were not additive to the effects of mobility-related state anxiety on the locomotor system. Speech pause duration and number were affected by motor complexity but were seemingly unaffected by the virtual mobility threat. Data suggest that subjective feelings of worry and confidence during the task, along with informal debriefing, may buffer healthy individuals from the deleterious effects of anxiety on mobility through distraction.

### Supplementary Information

Below is the link to the electronic supplementary material.

Supplementary file1 (DOCX 40 KB)

## Data Availability

Data available upon request.
